# Stability of RNA quadruplex in open reading frame determines proteolysis of human estrogen receptor α

**DOI:** 10.1093/nar/gkt286

**Published:** 2013-04-24

**Authors:** Tamaki Endoh, Yu Kawasaki, Naoki Sugimoto

**Affiliations:** ^1^Frontier Institute for Biomolecular Engineering Research (FIBER), Konan University, 7-1-20 Minatojimaminamimachi, Kobe 650-0047, Japan and ^2^Faculty of Frontiers of Innovative Research in Science and Technology (FIRST), Konan University, 7-1-20 Minatojimaminamimachi, Kobe 650-0047, Japan

## Abstract

mRNAs encodes not only information that determines amino acid sequences but also additional layers of information that regulate the translational processes. Notably, translational halt at specific position caused by rare codons or stable RNA structures is one of the potential factors regulating the protein expressions and structures. In this study, a quadruplex-forming potential (QFP) sequence derived from an open reading frame of human estrogen receptor α (*hERα*) mRNA was revealed to form parallel G-quadruplex and halt the translation elongation *in vitro*. Moreover, when the full-length *hERα* and variants containing synonymous mutations in the QFP sequence were expressed in cells, translation products cleaved at specific site were observed in quantities dependent on the thermodynamic stability of the G-quadruplexes. These results suggest that the G-quadruplex formation in the coding region of the *hERα* mRNA impacts folding and proteolysis of hERα protein by slowing down or temporarily stalling the translation elongation.

## INTRODUCTION

During translation elongation, the ribosome decodes mRNA sequence and converts the information into the amino acid sequence of protein. Ribosome progression is arrhythmic rather than uniform because of features of mRNAs. Rare codons and specific secondary structures in open reading frames (ORFs) are known to slow or stall translation elongation (1,2). Previous studies have revealed that stalling of translation elongation at specific position regulates protein expression by affecting translation initiation of a second ORF or by inducing ribosomal frameshifts (2–5). In addition, the positions of rare codons and stable secondary structures of mRNA correlate with flexible linker regions in protein structures (6–9). As proteins partially fold during translation elongation (10–12), the rare codons and secondary structures of mRNA likely affect the structures of nascent proteins (8,9,13–18). These observations suggest that mRNA sequence and structure might impact not only expression levels but also the processing of proteins.

G-quadruplex is one of the non-canonical structures formed by guanine-rich (G-rich) sequence in DNA and RNA stabilized by stacking of planes consisting of four guanine bases that interact through Hoogsteen-type hydrogen bonds (19,20). The G-quadruplexes formed by RNAs are generally more stable than those formed by equivalent DNA sequences (21–23). Our previous studies indicate that stabilities of G-quadruplexes are higher in the presence of high concentration of cosolute, which might mimic cell-like molecular crowding condition, and encapsulated conditions than in diluted solution (24,25), whereas canonical secondary structures are destabilized in the presence of cosolute, especially solutes decreasing water activity (26,27). In addition, we recently performed that RNA G-quadruplex formed in ORF of mRNA suppressed translation elongation *in vitro* and reduced protein expression in cells (28). Thus, the G-rich sequence conserved in mRNAs likely forms RNA G-quadruplex in cells and contributes to biological procedures exhibiting an effect as strong as the canonical secondary structures based on Watson–Crick-type hydrogen bonds.

**Figure 1.**
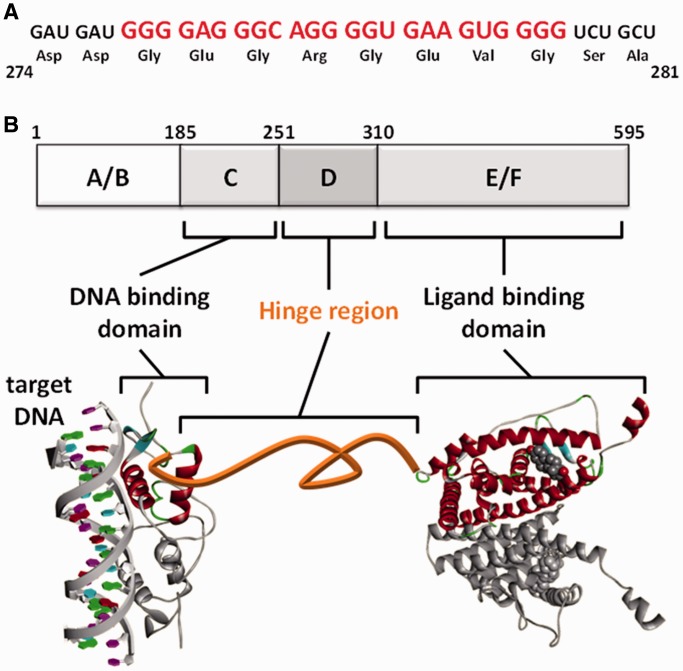
QFP sequence derived from *hERα* mRNA. (**A**) QFP sequence in the ORF of *hERα* mRNA. Amino acids coded on the QFP sequence are indicated. (**B**) Domains of hERα protein. The hinge region (domain D), which contains the QFP sequence, connects the DNA-binding domain (domain C) and ligand-binding domain (domain E/F). Structures of the DNA-binding domain (PDB: 1HCQ) and ligand-binding domain (PDB: 1A52) are shown.

Human estrogen receptor α (hERα) is a ligand-inducible nuclear transcription factor that activates expression of genes responsible for growth, development and maintenance of the reproductive, skeletal, neuronal and immune systems in response to estrogens (29,30). The *hER*α mRNA contains a sequence with quadruplex-forming potential (QFP) in the region of the ORF that codes for domain D, a hinge region of the protein (Figure 1A) (29,31). Preceding DNA-binding domain (domain C) and after ligand-binding domain (domain E/F) form respective domain structures and contribute the ligand-dependent gene expressions (Figure 1B). If the QFP sequence adopts a G-quadruplex structure and slows or stalls the translation elongation, it might affect the co-translational folding of the domain structures (Figure 1B). In this study, we investigated effects of the QFP sequence derived from the *hER*α mRNA on protein expression.

## MATERIALS AND METHODS

### Preparation of RNA

RNA oligonucleotides used in this study were purchased from Japan Bio Services Co., Ltd and were purified by high-performance liquid chromatography (HPLC). Concentrations of the RNA oligonucleotides were determined by measurement of the absorbance at 260 nm at a high temperature using a Shimadzu-1800 ultraviolet (UV)/Vis spectrophotometer connected to a thermoprogrammer. Extinction coefficients were calculated from mononucleotide and dinucleotide data using nearest-neighbor approximations.

Reporter mRNAs were prepared by *in vitro* transcription. A DNA template for transcription of a reporter mRNA without the QFP sequence was amplified by polymerase chain reaction (PCR) from a plasmid vector, previously constructed for synchronized translation (32) using a T7 promoter primer and an antisense primer. This DNA has the sequence 5′-TAAATGAATTTTTTTATTAATAAATAAGATTTCATAGAAAGCATTTTGT-3′. DNA templates for reporter mRNAs containing QFP sequence variants were amplified from the first PCR product by using T7 promoter primer and the respective antisense primer. Antisense primers containing wild-type, A-mutant, C-mutant, G-mutant, and U-mutant QFP sequences are 5′-TTTTTTAGACCCCACTTCACCCCTGCCCTCCCCATCTTTACCTAAATGAATTTTTTTATTAATAAATAA-3′, 5′-TTTTTTAGATCCTACTTCACCTCTGCCTTCTCCATCTTTACCTAAATGAATTTTTTTATTAATAAATAA-3′, 5′-TTTTTTAGACCCGACTTCACCCCTGCCCTCCCCATCTTTACCTAAATGAATTTTTTTATTAATAAATAA-3′, 5′-TTTTTTAGACCCCACTTCCCCCCTCCCCTCCCCATCTTTACCTAAATGAATTTTTTTATTAATAAATAA-3′, and 5′-TTTTTTAGAACCAACTTCACCCCTGCCCTCACCATCTTTACCTAAATGAATTTTTTTATTAATAAATAA-3′, respectively. mRNAs were prepared by using ScriptMAX Thermo T7 Transcription Kit (ToYoBo) and purified on denaturing polyacrylamide gel. All the mRNAs were refolded by cooling from 90°C to 4°C at 1°C min^−1^ in a buffer containing 30 mM HEPES (pH 6.8) and 100 mM KCl.

### UV measurements

UV absorbance was measured with a Shimadzu-1800 UV/Vis spectrophotometer equipped with a temperature controller. Melting curves of 5 μM RNA oligonucleotides were obtained by measuring the UV absorbance at 260 and 295 nm in a buffer containing 50 mM MES–LiOH (pH 7.0) and 3 mM KCl in the presence or absence of 40 wt% PEG200. The heating rate was 0.2°C min^−^^1^. The thermodynamic stabilities at 37°C (Δ*G°*_37_) were calculated from the fit of the melting curves to a theoretical equation for an intramolecular association as described previously (25,33). Before the measurement, the samples were heated to 95°C, cooled at a rate of 0.2°C min^−^^1^ and incubated at 4°C for 10 min.

### Non-denaturing polyacrylamide gel electrophoresis

RNA oligonucleotide (5 µM) was heated to 95°C and cooled to 4°C at a rate of 0.2°C min^−^^1^ in a buffer containing 50 mM MES–LiOH (pH 7.0), 3 mM KCl and 40 wt% PEG200. Aliquots (10 pmol) were separated on a 15% polyacrylamide gel containing K3-TB buffer (89 mM Tris, 89 mM borate and 3 mM KCl) at 37°C. K3-TB was used as a running buffer. After gel electrophoresis, RNA oligonucleotides were stained by ethidium bromide, and gels were imaged using FLA5100 fluorescence image scanner (Fuji Film) with 532 nm excitation and 575 nm emission.

### CD measurements

After UV measurements in a buffer containing 50 mM MES–LiOH (pH 7.0) and 3 mM KCl in the presence or absence of 40 wt% PEG200, RNA oligonucleotides were cooled to 20°C at a rate of 1°C min^−^^1^. Circular dichroism (CD) spectra were collected on a JASCO J-820 spectropolarimeter at 37°C in 1.0-mm path length cuvettes. The CD spectra shown are averages of three scans. The temperature of the cell holder was regulated by a JASCO PTC-348 temperature controller.

### Synchronized translation

Synchronized translation was performed according to a previously published experimental procedure (32) with slight modification. An *in vitro* translation mixture (PUREfrex; GeneFrontier) without release factor (RF)1, RF2 and tyrosyl-tRNA synthetase (TyrRS) was pre-incubated for 10 min. An amber suppressor tRNA (CR110-X-AF-tRNAamber; Protein Express) was added at final concentration of 1.6 μM. The first step was started by addition of reporter mRNAs at a final concentration of 3.6 μM. Simultaneously with the first reaction, reaction mixture of PUREfrex containing all requisite proteins except ribosomes was pre-incubated. After 10 min, the second step was started by addition of an equal volume of the pre-incubated reaction mixture to the reaction mixture of the first reaction. Aliquots of the reaction mixture were quickly frozen in liquid nitrogen at indicated times. Both of steps were performed at 37°C using tRNA concentrations specified by the manufacturer; the absorbance at 260 nm of the tRNA mix (A260/ml tRNAmix) was 20. Reaction mixtures were separated on a 16% Tris–tricine sodium dodecyl sulfate–polyacrylamide gel electrophoresis (SDS–PAGE) (34). Before electrophoresis, an equal volume of 4 M urea was added to the reaction mixture at 4°C followed by an addition of RNase A and incubation at 37°C for 10 min. Fluorescence signals of CR110-X-AF on the gel were imaged by FLA5100 with 473 nm excitation and 510 nm emission.

### Mass spectrometry analysis

Reaction mixtures treated with RNase A were purified using anti-T7-tag agarose (MBL) as described previously (32). The mass spectrum of the purified products was analyzed by using a MALDI-TOF mass spectrometer (Autoflex III; Bruker Daltonics).

### Construction of plasmids

Total RNA was purified from MCF7 cells using an RNeasy kit (Qiagen). A cDNA library was constructed from the RNA by reverse transcription using oligo-dT primer. The coding region of wild-type *hER*α was amplified by PCR and cloned into BamHI and XhoI sites of pcDNA5/FRT (Invitrogen, USA) to construct phERα. Coding sequences of *hERα* containing the QFP sequence variants were constructed by two-step PCR and cloned into same sites of restriction enzymes to construct phERα variants. All primers are listed in Supplementary Table S1. DNA fragments of *hERα* containing QFP sequence variants were digested by BamHI and XhoI and ligated into BglII and SalI sites of pAcGFP-C1 and pAcGFP-N3 (Clontech) to make pAcGFP–hERα and phERα–AcGFP, respectively.

### Cell culture

Media for culturing MCF7, HepG2 and Flp-In 293 cells were Eagle's minimum essential medium containing non-essential amino acids (EMEM), EMEM and Dulbecco’s modified Eagle’s medium, respectively. Media was supplemented with 10% fetal bovine serum and antibiotics (penicillin 100 U/ml and streptomycin 100 μg/ml). Cells were maintained at 37°C under 5% CO_2_.

### Western blotting

HepG2 cells were plated on collagen-coated six-well plates at a density of 5 × 10^5^ cells per well and incubated overnight. One microgram of phERα containing the QFP sequence variants was transfected into HepG2 cells using 6 μl of X-treamGENE 9 (Roche) according to the manufacturer’s protocol. After incubation at 37°C for 48 h, cells were harvested and lysed by sonication (total of 1.5 min) in a lysis buffer containing 0.2% Nonidet P-40 (NP-40) and 0.05% SDS, and cellular debris were removed by centrifugation. Aliquots of cell lysates were separated on a 10% SDS–PAGE, and hERα was detected by western blotting using an anti-hERα mouse monoclonal antibody (Clone 1D5; Thermo Scientific) and anti-mouse IgG antibody conjugated with alkaline phosphatase (Promega).

### Fluorescence imaging of AcGFP

MCF7, HepG2 and Flp-In 293 cells were plated on collagen-coated 24-well plate at densities of 5 × 10^4^, 1 × 10^5^ and 1 × 10^5^ cells per well, respectively, and incubated overnight. Cells were transfected with 250 ng of pAcGFP–hERα or phERα–AcGFP containing the QFP sequence variants using 1.5 μl of X-treamGENE 9 according to the manufacturer’s protocol. After incubation at 37°C for 48 h, cells were lysed by sonication (total of 1.5 min) in a lysis buffer containing 0.2% NP-40 and 0.05% SDS, and cellular debris were removed by centrifugation. Aliquots of cell lysates were separated on an 8% SDS–PAGE without denaturing. Fluorescence signals were imaged by FLA5100 with 473 nm excitation and 510 nm emission, and signal intensities were calculated by Multi Gauge software (Fuji Film).

## RESULTS

### G-quadruplex formation by *hERα* mRNA sequence

**Figure 2.**
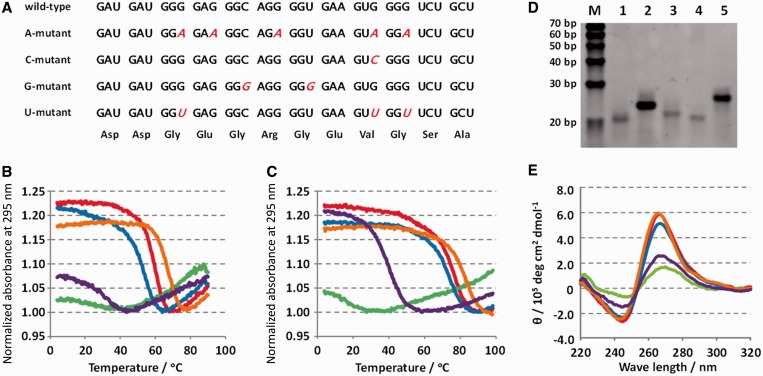
Formation of G-quadruplexes by QFP sequence variants. (**A**) RNA oligonucleotides corresponding to the QFP sequence derived from ORF of *hERα* mRNA and variants that mutate the G-rich regions (indicated in red and italic). Amino acid sequences encoded by the QFP sequence variants are indicated. All mutations are synonymous. (**B** and **C**) Normalized absorbance at 295 nm of 5 μM QFP sequence variants, wild-type (red), A-mutant (green), C-mutant (blue), G-mutant (orange) and U-mutant (purple), in a buffer containing 50 mM MES–LiOH, pH 7.0, and 3 mM KCl in the absence (B) and presence (C) of 40 wt% PEG200. (**D**) Non-denaturing 12% PAGE of QFP sequence variants. Ten picomoles of wild-type (lane 1), A-mutant (lane 2), C-mutant (lane 3), G-mutant (lane 4) and U-mutant (lane 5) was incubated at 95°C and cooled to room temperature at 0.2°C min^−1^ before loading. Electrophoresis was performed at 37°C with 3 W of constant electricity. Lane M shows 10 bp ladder marker. (**E**) CD spectra of 5 μM QFP sequence variants, wild-type (red), A-mutant (green), C-mutant (blue), G-mutant (orange) and U-mutant (purple), at 37°C in a buffer containing 50 mM MES–LiOH, pH 7.0, 3 mM KCl and 40 wt% PEG200.

RNA oligonucleotides corresponding to the QFP sequence derived from the ORF of the *hERα* mRNA and variants containing mutations that encode the same amino acid sequence (Figure 2A) were synthesized, and their thermodynamic stabilities and structures were evaluated *in vitro*. As the QFP sequences had melting temperature higher than 90°C in the presence of a physiological concentration (100 mM) of potassium (data not shown), UV melting data were obtained in buffer containing 3 mM potassium. Figure 2B shows normalized UV melting profiles of the QFP sequence variants analyzed from absorbance at 295 nm. Wild-type, C-mutant and G-mutant oligonucleotides had curves typical of G-quadruplex. The A-mutant and U-mutant oligonucleotides showed melting transitions at 260 nm both in the presence of 3 and 100 mM potassium (Supplementary Figure S1A), suggesting formation of secondary structures containing Watson–Crick-type base pairs in diluted solution as predicted by the mfold program (Supplementary Figure S1B) (35). However, the U-mutant also showed little melting transition at 295 nm in the presence of 100 mM potassium, although the melting temperature at 295 nm seemed less than that at 260 nm (Supplementary Figure S1C). This transition indicates that the U-mutant potentially forms G-quadruplex. To focus on the stability of G-quadruplexes formed by the QFP sequence variants, the UV melting profiles were recorded in the presence of 40 wt% polyethylene glycol (PEG) having averaged molecular weight of 200 (PEG200) as a cosolute because PEG200 has previously shown to stabilize quadruplexes but destabilize duplexes (21,25–27). Figure 2C shows normalized UV melting profiles of the QFP sequence variants at 295 nm in the presence of 3 mM potassium and 40 wt% PEG200. The melting temperatures of wild-type, C-mutant and G-mutant oligonucleotides shifted to higher temperatures under these conditions. The U-mutant also showed a clear melting transition at 295 nm. The U-mutant G-quadruplex may have mix-tetrad consists of guanines and uracils like U-tetrad shown to stabilize the human telomeric RNA G-quadruplex (Supplementary Figure S2) (36). When analyzed by native PAGE at 37°C, all the QFP sequence variants migrated as single bands (Figure 2D). Wild-type, C-mutant and G-mutant oligonucleotides migrated faster than other two mutants, suggestive of intramolecular G-quadruplex structures. The weak signal intensities of wild-type, C-mutant and G-mutant compared with the U-mutant and A-mutant signals likely reflect poorer binding of ethidium bromide to the quadruplexes.

CD spectra of the QFP sequence variants at 37°C were also obtained in the presence of 3 mM potassium and 40 wt% PEG200 (Figure 2E). Wild-type, C-mutant, G-mutant and U-mutant spectra were characterized by positive and negative peaks near 265 and 240 nm, respectively, which were larger than those of observed in the A-mutant spectra. These results indicated that all QFP sequence variants with the exception of the A-mutant formed parallel G-quadruplex structures. Wild-type, C-mutant, G-mutant and U-mutant indicated melting transitions at 265 nm that corresponded to the UV melting transitions in Figure 2C (Supplementary Figure S3A). In addition, the CD spectra had isosbestic point near 250 nm and linear correlation between signal intensities at 265 and 242 nm during the melting transition (Supplementary Figure S3B). Thus, the RNA G-quadruplexes formed by the QFP sequence variants ware suggested to be melted by two-state transition. CD spectra of wild-type, C-mutant and G-mutant were virtually identical in the presence and absence of 40 wt% PEG200 (Supplementary Figure S4). The monophonic properties of RNA G-quadruplex irrespective of solution conditions (21,37) might be a cause of the two-state transitions. Thermodynamic stabilities at 37°C (Δ*G°*_37_) and melting temperatures (*T*m) of the QFP sequence variants in the presence of 3 mM potassium and 40 wt% PEG200 were calculated from the UV melting profiles by van’t Hoff equation (Table 1).

**Table 1.** Thermodynamic stabilities of QFP sequence variants in the presence of 3 mM potassium and 40 wt% PEG200

QFP sequence variants	Δ*G°*_37_^a^/kcal mol^−1^	*T*m/°C
wild-type	−5.15 ± 0.84	76.4 ± 0.9
A-mutant	ND	ND
C-mutant	−4.77 ± 0.37	72.6 ± 1.2
G-mutant	−5.86 ± 0.55	82.4 ± 1.0
U-mutant	−0.47 ± 0.03	39.9 ± 0.5

^a^Values of Δ*G°*_37_ were calculated from five melting profiles of the QFP sequence variants at the heating rate of 0.2°C min^−1^.

### Suppression of translation by QFP sequence

**Figure 3.**
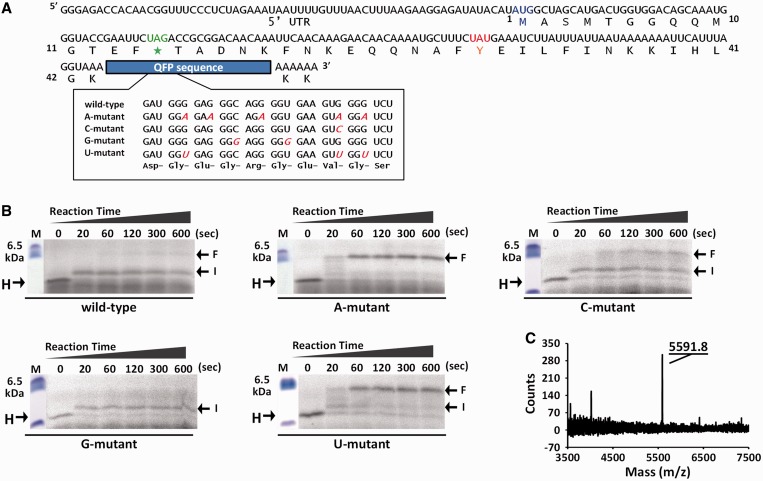
Translational halt before QFP sequence *in vitro*. (**A**) Nucleotide and amino acid sequences of reporter mRNAs containing QFP sequence variants derived from *hERα*. Positions of start codon (blue), amber codon for incorporation of non-natural fluorescent amino acid (green, asterisk) and tyrosine codon to artificially halt translation (orange) are indicated. Nucleotides mutated relative to wild-type QFP sequence are indicated in red. (**B** and **C**) Time course of the synchronized translation of reporter mRNAs. Samples taken from the synchronized translation at indicated reaction times were resolved on 16% Tris–tricine SDS–PAGE. The fluorescence signal of incorporated non-natural amino acid (CR110-X-AF) was imaged using 473 nm excitation and 510 nm emission. Translated products because of ribosomal stalling (I) and complete elongation (F) are indicated. (**D**) Mass spectrometry analysis of translated products of wild-type mRNA after 20 s of synchronized translation. Observed mass of main signal is indicated.

To evaluate the effect of the G-quadruplex formed by the QFP sequence derived from the *hERα* mRNA on translation elongation, reporter mRNAs containing the QFP sequence variants at 3′ terminal of the mRNA were constructed (Figure 3A). Formation of G-quadruplex in the mRNAs was confirmed by using fluorescence signal of *N*-methyl mesoporphyrin (NMM), which specifically binds to G-quadruplex structure and increase its fluorescence (Supplementary Figure S5A) (38). Higher fluorescence intensities with wild-type, C-mutant, G-mutant and U-mutant mRNAs than that with A-mutant mRNA or without mRNA suggest formation of G-quadruplexes in the reporter mRNAs containing wild-type and mutant QFP sequences except A-mutant. It should be noted that the mRNAs were refolded in the presence of 100 mM potassium and absence of PEG200 because PEG200 would disturb *in vitro* translation (39). The U-mutant mRNA was considered to partially formed G-quadruplex as expected from UV melting profile (Supplementary Figure S1C) and slightly increased the fluorescence signal of NMM.

Time course of translation elongation was analyzed *in vitro* by using a synchronized translation that we recently developed to evaluate a single turnover of the elongation reaction (32). In the first step of the reaction, translation was performed in a reconstructed *in vitro* translation system (PUREfrex; GeneFrontier) in which RF1, RF2 and TyrRS were left out of the reaction mixture; this artificially halted translation before the 30th codon encoding tyrosine (Figure 3A). During this reaction, a non-natural fluorescent amino acid (CR-110-X-AF) was incorporated into the protein by using an amber suppressor tRNA aminoacylated with CR110-X-AF (CR110-X-AF-tRNAamber; Protein Express). In the second step, translation elongation was restarted by addition of a reaction mixture containing all requisite proteins except the ribosome. This second reaction was stopped at 20, 60, 120, 300 and 600 s from the restart. Fluorescence signal of CR110-X-AF, which was incorporated into translated product, was imaged after separation by Tris–tricine SDS–PAGE. Figure 3B shows the time course of the translation elongation using the reporter mRNAs containing QFP sequence variants. Strong signals near the bottom of the gel are due to unincorporated CR110-X-AF (32). Before the restart, the fluorescence signal of a 29-residue peptide because of the artificial halt of translation before the tyrosine codon was similar in reaction with all the reporter mRNAs (Figures 3B, indicated by H). In the case of wild-type, C-mutant and G-mutant mRNAs, which form relatively stable G-quadruplexes, the main translation products were observed at a slightly higher position (Figure 3B, indicated by I) comparing with the product before the restart. The fluorescence signal indicated by I corresponds translation product in which translation elongation was stalled at an intermediate position of mRNA because the main translation products with U-mutant and A-mutant mRNAs were observed at further higher position (Figure 3B, indicated by F) despite the amino acid sequence encoded by all of the reporter mRNAs are the same (Figure 3A). The fluorescence signal indicated by F corresponds the final product in which the translation elongation progressed to the 3′-end of the mRNA. The intermediate product indicated by I was temporarily observed at 20 s from the restart in the case of the U-mutant mRNA, and the signal was gradually disappeared with increasing the reaction time. In addition, the final product indicated by F was gradually increased with increasing the reaction time in the case of wild-type and C-mutant mRNAs. From these results, it was suggested that the RNA G-quadruplex formed in the reporter mRNAs stalled the translation elongation, and time range of the stalling depends on the stability of the RNA G-quadruplex (Table 1).

To determine the position, where the translation elongation was stalled, the mass spectrum of the reaction at 20 s after the translation restart was analyzed using wild-type mRNA (Figure 3D). The signal at 5591.8 kDa corresponded to the mass of a 42-residue peptide with an adenosine moiety at the C terminus (calculated mass was 5592.2 with protonation). The position of the 42nd codon corresponds to a site on the mRNA six nucleotides before the QFP sequence. The stalling position corresponded to the position that we observed using mRNA-containing RNA G-quadruplexes derived from *Escherichia coli* genes (28). The mass of the 42-residue peptide was not observed in the translation product with A-mutant mRNA (data not shown). These data also indicated that translation elongation was specifically stalled by the G-quadruplex structure formed on the mRNA that block the mRNA entry into ribosome.

### hERα expression depends on QFP sequence

The potent translation suppression mediated by the RNA G-quadruplex formed by QFP sequence of *hERα* mRNA may impact *hERα* expression inside cells. The coding sequence of *hERα* was cloned into plasmid vector, and variants containing synonymous mutations in the QFP sequence were constructed. To confirm whether the QFP sequence in natural *hERα* mRNA form RNA G-quadruplex, as well as reporter mRNA, full-length mRNA encoding *hERα* variants were prepared by *in vitro* transcription and mixed with NMM (Supplementary Figure S5B). The results were similar to those of the reporter mRNAs for synchronized translation. Although slightly higher fluorescence signal with A-mutant *hERα* mRNA compared with that without mRNA made it difficult to find significant difference between the A-mutant and U-mutant *hERα* mRNAs, other three mRNAs containing wild-type, C-mutant and G-mutant QFP sequences were confirmed to form G-quadruplex because the fluorescence intensities were further increased comparing with the A-mutant and U-mutant *hERα* mRNAs. It is considered that NMM non-specifically bound to the A-mutant *hERα* mRNA because the length of the *hERα* mRNAs (1946 nt) was much longer than that of reporter mRNAs (228 nt) used in Supplementary Figure S5A.

**Figure 4.**
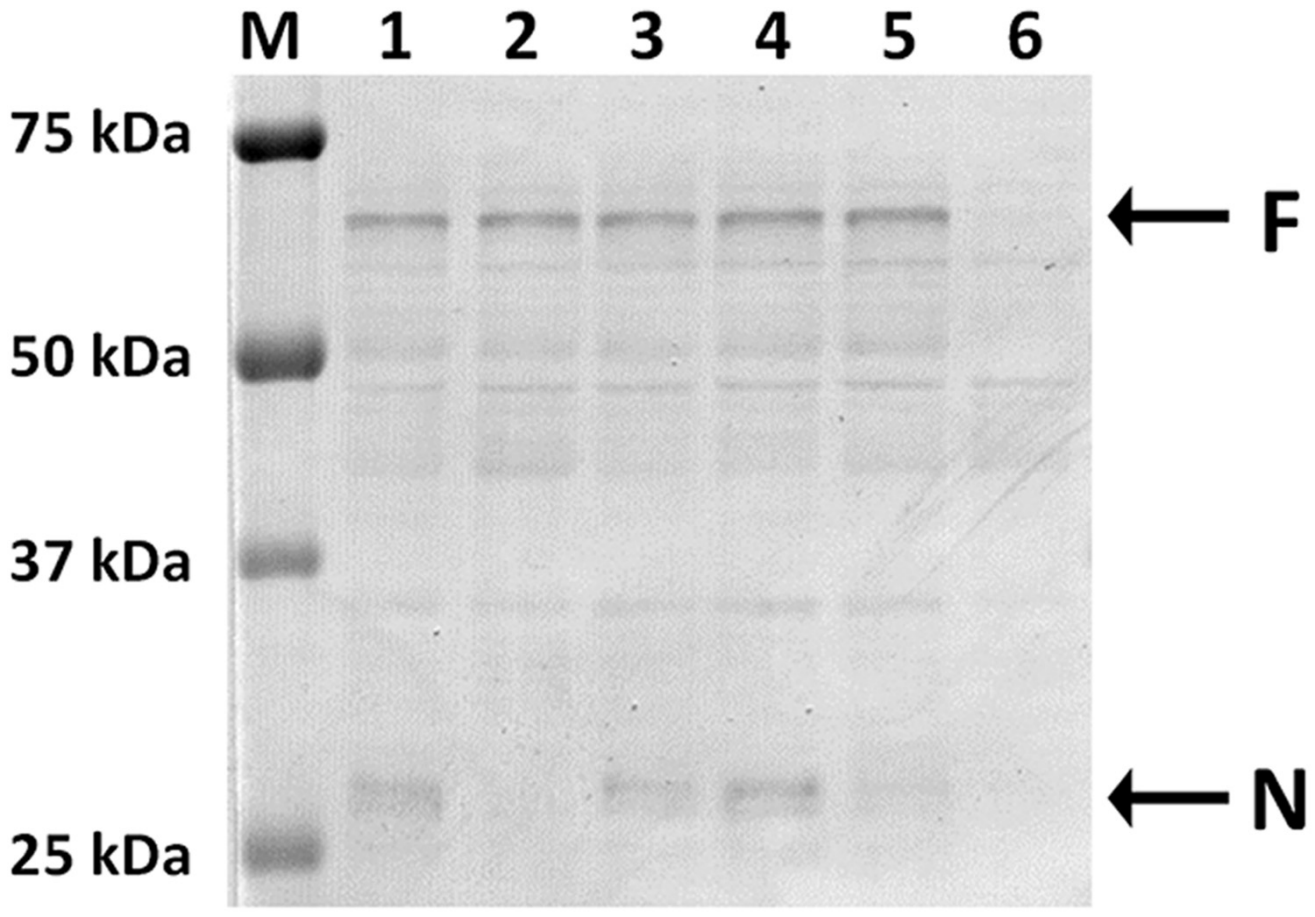
Expression of hERα in HepG2 cells. Vectors for expression of hERα containing the QFP sequence variants, wild-type (lane 1), A-mutant (lane 2), C-mutant (lane 3), G-mutant (lane 4) and U-mutant (lane 5), or control plasmid (pcDNA5/FRT, Invitrogen) (lane 6) were transfected into HepG2 cells. Cell lysates obtained after 48-h incubation at 37°C were resolved on 8% SDS–PAGE. Expression of hERα was analyzed by western blotting using an anti-hERα antibody specific for the N-terminal domain. Lane M shows marker proteins. Signals of hERα correspond to full-length protein, and shorter forms are indicated by F and N, respectively.

The plasmid vectors were transfected into HepG2 human liver carcinoma cells in which the expression of endogenous hERα is extremely low (40). Figure 4 shows results of western blotting using anti-hERα antibody specific for N-terminal domain of the protein (Thermo Scientific). It should be noted that the hERα expressed from the vectors contains an additional seven amino acids at C-terminus relative to the endogenous form, and the full-length hERα obtained from the expression vectors should be 67 kDa. In cells transfected with all the QFP sequence variants, a signal corresponding to full-length hERα was observed located between the 50- and the 75-kDa marker proteins (Figure 4, indicated by F). In cells transfected with wild-type and G-mutant, a strong signal was observed from a band that migrated slightly slower than the 25-kDa marker protein (Figure 4, indicated by N). Weaker signals from this region were also observed in C-mutant and U-mutant transfected cells, whereas the signal was vanishingly low in A-mutant transfected cells. The amino acid sequence of hERα expressed from each of these vectors is the same. As the signal intensities of the short hERα correlated with the thermodynamic stability of the G-quadruplex formed by the QFP sequence variants (Table 1), the production of the short hERα was likely caused by the differences in the G-quadruplexes formed within the ORF of mRNAs.

As shown in Figure 3, if the translation elongation was stalled before the QFP sequence and truncated at the position during the stalling, the product would include 273 amino acids and have a molecular weight of 30.0 kDa, which is close to that of the product observed on the gel (Figure 4 indicated by N). The G-quadruplex formed by the QFP sequence might cause the production of the short hERα by inducing an mRNA surveillance mechanism termed no-go mRNA decay, which was recently discovered in yeast and is thought to be caused by an artificially introduced stable duplex (41,42). A previous study demonstrated that the hERα translated *in vitro* using rabbit reticulocyte and wheat germ extracts exhibited different properties against proteolysis by chymotrypsin (43). This observation suggests an alternative possibility that the production of the short hERα might due to the proteolysis of the full-length product.

### Proteolysis of hERα depends on QFP sequence

**Figure 5.**
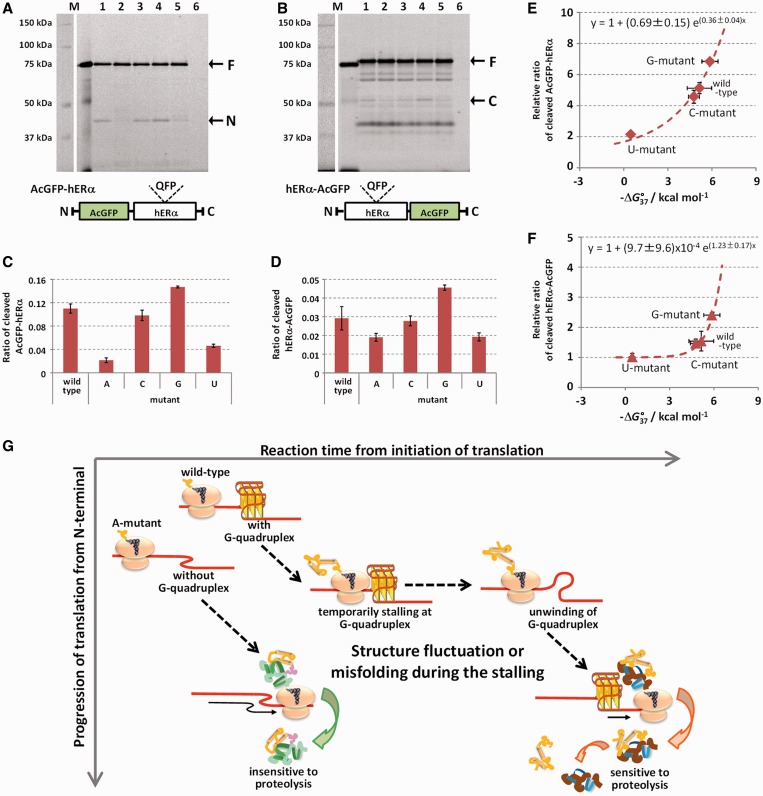
Proteolysis of hERα depending of thermodynamic stabilities of G-quadruplexes. (**A** and **B**) Protein expression patterns of AcGFP–hERα (A) and hERα–AcGFP (B) in MCF7 cells. Cells were transfected with vectors for expression of AcGFP–hERα or hERα–AcGFP containing the QFP sequence variants, wild-type (lane 1), A-mutant (lane 2), C-mutant (lane 3), G-mutant (lane 4) and U-mutant (lane 5) or were not transfected with plasmid (lane 6). Cells were lysed after 48-h. Cell lysates were resolved on 8% SDS–PAGE without denaturing, and fluorescence signals were imaged using 473 nm excitation and 510 nm emission. (**C** and **D**) Ratio of cleaved AcGFP–hERα (C) and hERα–AcGFP (D) relative to the full-length protein (*n* = 3). (**E** and **F**) Plots of the relative ratios of the cleaved AcGFP–hERα (E) and hERα–AcGFP (F) normalized to the ratio of A-mutant versus −Δ*G°*_37_ of the QFP sequence variants calculated from the UV melting. Fits to a single exponential equation (interior and dashed line) are indicated. Error bars represent SD. (**G**) Scheme of translation procedures of wild-type mRNA (with G-quadruplex) and A-mutant mRNA (without G-quadruplex). Temporarily stalling of the ribosome before the G-quadruplex affects co-translational folding of nascent protein and makes the protein structure sensitive to proteolysis.

To quantitatively evaluate the effect of the G-quadruplex stabilities on the production of the short hERα, the coding sequence of the fluorescent protein (AcGFP) was inserted either into the 5′ or the 3′ region of hERα to express AcGFP fused to hERα at the N-terminus (AcGFP–hERα) or the C-terminus (hERα–AcGFP), respectively. Vectors were transfected into MCF7 human breast carcinoma cells that consecutively express hERα. AcGFP preserves its fluorescence during SDS–PAGE unless it has been denatured before the electrophoresis. Figure 5A and B show fluorescence signals of AcGFP–hERα and hERα–AcGFP, respectively, in 8% SDS–PAGE. AcGFP–hERα showed similar expression patterns when analyzed by western blotting. Fluorescence signals of the full-length protein were observed near the 75-kDa marker protein in all the QFP sequence variants, and for certain QFP sequence variants a signal was observed between the 37- and the 50-kDa maker proteins (Figure 5A, indicated by N). In the case of hERα–AcGFP, although several signals appeared uniformly in all the QFP sequence variants, a fluorescence signal near the 50-kDa marker was observed for certain QFP sequence variants (Figure 5B, indicated by C). Because hERα–AcGFP contains AcGFP at C-terminus, the fluorescence signal depending on the QFP sequence variants would not be observed if the cause of the short hERα observed in Figure 4 was truncation of translation during the stalling of the translation elongation. Thus, the expression patterns of both AcGFP–hERα and hERα–AcGFP suggested that the short hERα results from proteolysis.

Ratios of the cleaved AcGFP–hERα (Figure 5A, indicated by N) and hERα–AcGFP (Figure 5B, indicated by C) relative to full-length protein (Figure 5A and B, indicated by F) were calculated from the fluorescence intensities (Figure 5C and D). Both ratios depended on the thermodynamic stabilities of the G-quadruplex formed by the QFP sequence variant; the variants able to form more stable G-quadruplexes resulted in more production of the short hERα. The ratios calculated for the A-mutant were used as standards, because the A-mutant did not form G-quadruplex even in the presence of 100 mM potassium and 40 wt% PEG200 (data not shown). Figure 5E and F show plots of relative ratio values of QFP sequence variants against the ratio value of A-mutant versus the thermodynamic stabilities of the QFP sequence variants calculated from UV melting transition in Figure 2B (Table 1). The data from both the cleaved AcGFP–hERα and hERα–AcGFP fit a single exponential function. In HepG2 cells and in cells from a human embryonic kidney cell-line (Flp-In 293, Invitrogen), the relative ratios of cleaved AcGFP–hERα also fit a single exponential function (Supplementary Figure S6).

## DISCUSSION

During translation elongation, the ribosome must unwind any structure formed by an mRNA. The mRNA structures and stabilities would affect both efficacy and velocity of the translation elongation. In the case of G-quadruplexes, it was recently suggested that G-quadruplex formation in 5′-untranslated region (UTR) of mRNAs potentially suppress protein expression level by inhibiting small ribosomal subunit in scanning, especially in the UTRs of oncogene mRNAs (44–47). The effect of the G-quadruplexes in ORFs of mRNAs has not been studied, although it has been speculated that suppression of elongation might impact folding and processing of nascent proteins (13,15). The QFP sequence in the ORF of the *hERα* mRNA is located in the region that encodes the hinge between the DNA-binding domain and the ligand-binding domain (Figure 1) (29). It was previously shown that proteolysis of hERα by chymotrypsin depends on the *in vitro* translation system used (43). Thus, structure and processing of hERα are likely to be affected if the QFP sequence forms a G-quadruplex and inhibits ribosome in elongation reaction.

The QFP sequence derived from *hERα* mRNA was cloned into 3′ region of reporter mRNA, and effects of the QFP sequence on translation elongation were evaluated in a synchronized translation system (Figure 3). Translation elongation was suppressed at six nucleotides before the wild-type QFP sequence. The parallel G-quadruplex formed by the QFP sequence (Figure 2) likely inhibits the helicase activity of ribosome (48) and blocks mRNA entry into the ribosome. The translation suppression was temporary rather than permanent because a full-length product was observed with increasing the reaction time depending on the thermodynamic stabilities of RNA G-quadruplexes formed by the QFP sequence variants (Figure 3B).

Analysis of expression of hERα in HepG2 cells indicated production of short hERα with a molecular weight ∼25–30 kDa that was recognized by anti-hERα antibody specific for N-terminal domain (Figure 4). The amount of short hERα produced depended on the synonymous mutations in the QFP sequence; none of this form was observed on translation of the A-mutant that could not form G-quadruplex. In addition, expression of mRNAs encoding AcGFP–hERα and hERα–AcGFP yielded products shorter than full-length protein in amounts that depended on the QFP sequence variants (Figure 5 and Supplementary Figure S5). These results indicated that the QFP sequence contributed proteolysis of hERα at a specific position. The relative ratios of the short to full-length products depended on the thermodynamic stabilities of the QFP sequence variants (Figure 5E and F), as well as suppression of translation elongation *in vitro* (Figure 3B). The ribosome should completely unwind G-quadruplex into single strand and import the mRNA to continue the translation. In addition, the ribosome progresses three nucleotides in every translocation reaction, which occurs within 0.1 s (1). This progression would be enough to unwind G-quadruplex consisting of three quartets at a time, and intermediate states during dissociation of G-quadruplex would be negligible. Thus, we assumed that the activation energy necessary for the ribosome to continue the translation elongation is proportional to the thermodynamic stability of the structure. In this case, the rate to overcome the stalling should depend on the G-quadruplex stability in a single exponential manner, and this is indeed what we observe. Similar results have been observed *in vitro* in correlation between mechanical strength of RNA pseudoknots and ribosomal frameshift, which is considered to be caused by temporary stalling of translation elongation before the pseudoknot (49). Thus, temporary stalling of translation elongation before the G-quadruplex (Figure 3B) is likely a main determinant of the proteolysis sensitivity of hERα by inducing fluctuation or misfolding of the nascent hERα (Figure 5G). The differences observed in the rabbit and wheat germ *in vitro* translation systems (43) might be due to differences in G-quadruplex stability in the two systems.

Although the proteolysis position has not been accurately determined, the short hERα had a molecular weight between 25 and 30 kDa (Figure 4, indicated by N). We assume that hERα is cleaved after the DNA-binding domain because the molecular weight of N-terminal 251 amino acids containing the activation function-1 domain (domain A/B) and the DNA-binding domain should be 27.6 kDa (29). The *hERα* pre-mRNA is known to be alternatively spliced resulting in hERα variants that confer dominant-positive and dominant-negative effects on the protein functions (50), and these variants are hypothesized to contribute to progression of certain cancers (51–54). The proteolysis products of wild-type hERα might retain certain functions, as well as products of the alternative splicing. In that case, the RNA G-quadruplex is considered to operate the protein polymorphism.

## SUPPLEMENTARY DATA

Supplementary Data are available at NAR Online: Supplementary Table 1 and Supplementary Figures 1–6.

## FUNDING

Grants-in-Aid for Scientific Research; MEXT-Supported Program for the Strategic Research Foundation at Private Universities (2009–2014), Japan. Funding for open access charge: Grants-in-Aid for Scientific Research, Japan.

*Conflict of interest statement*. None declared.

## Supplementary Material

Supplementary Data
