# Supplementary material for: Stability of RNA quadruplex in open reading frame determines proteolysis of human estrogen receptor α
Source: Nucleic Acids Res. 2013 Apr 24;41(12):6222–31. doi: 10.1093/nar/gkt286 (PMC3695533; doi:10.1093/nar/gkt286)
Supplement: Supplementary Data [file supp_41_12_6222__index.html]

Stability of RNA quadruplex in open reading frame determines proteolysis of human estrogen receptor α — Supplementary Data 

# Stability of RNA quadruplex in open reading frame determines proteolysis of human estrogen receptor α

## Supplementary Data

files

**Files in this Data Supplement:**

- Supplementary Data - pdf file
