# Supplementary material for: Stability of RNA quadruplex in open reading frame determines proteolysis of human estrogen receptor α
Source: Nucleic Acids Res. 2013 Apr 24;41(12):6222–31. doi: 10.1093/nar/gkt286 (PMC3695533; doi:10.1093/nar/gkt286)
Supplement: Supplementary Data [file supp_gkt286_Revised_Supplementary_Information_gkt286.pdf]

Supplementary Information

Stability of RNA quadruplex in open reading frame determines proteolysis of human estrogen receptor α

Tamaki Endoh,[1] Yu Kawasaki,[2] and Naoki Sugimoto[1,2] *

[1]Frontier Institute for Biomolecular Engineering Research (FIBER), Konan University, 7-1-20 Minatojimaminamimachi, Kobe, 650-0047, Japan

[2]Faculty of Frontiers of Innovative Research in Science and Technology (FIRST), Konan University, 7-1-20 Minatojimaminamimachi, Kobe, 650-0047, Japan

Email: sugimoto@konan-u.ac.jp

Table S1. Primers for PCR reaction

Primers for cloning wild-type *hERα*		DNA sequence
sense<br>antisense		ACCGGATCCATGACCATGACCCTCCACACC<br>TGGCTCGAGGACCGTGGCAGGGAAACCC
Primers for cloning *hERα* variants		
A-mutant	sense<br>antisense	GCGCCAGAGAGATGATGGAGAAGGCAGAGGTGAAGTAGGATCTGCTGGAGACATGAGAGC<br>GCTCTCATGTCTCCAGCAGATCCTACTTCACCTCTGCCTTCTCCATCATCTCTCTGGCGC
C-mutant	sense<br>antisense	GGAGGGCAGGGGTGAAGTCGGGTCTGCTGGAGACATGAG<br>CTCATGTCTCCAGCAGACCCGACTTCACCCCTGCCCTCC
G-mutant	sense<br>antisense	GAGATGATGGGGAGGGGAGGGGGGAAGTGGGGTCTGCTG<br>CAGCAGACCCCACTTCCCCCCTCCCCTCCCCATCATCTC
U-mutant	sense<br>antisense	CCAGAGAGATGATGGTGAGGGCAGGGGTGAAGTTGGTTCTGCTGGAGACATG<br>CATGTCTCCAGCAGAACCAACTTCACCCCTGCCCTCACCATCATCTCTCTGG

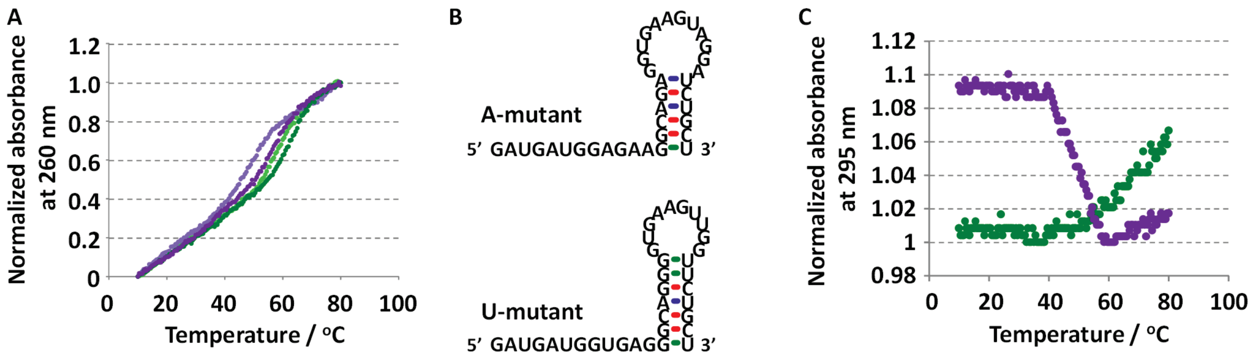

Figure S1. A) Normalized absorbance at 260 nm of 5 μM QFP sequence variants, A-mutant (green) and U-mutant (purple), in a buffer containing 50 mM MES-LiOH, pH 7.0, and 3 mM KCl (light colored) or 100 mM KCl (dark colored). B) Secondary structures of A-mutant and U-mutant predicted by mfold program. C) Normalized absorbance at 295 nm of 5 μM QFP sequence variants, A-mutant (green) and U-mutant (purple), in a buffer containing 50 mM MES-LiOH, pH 7.0, and 100 mM KCl.

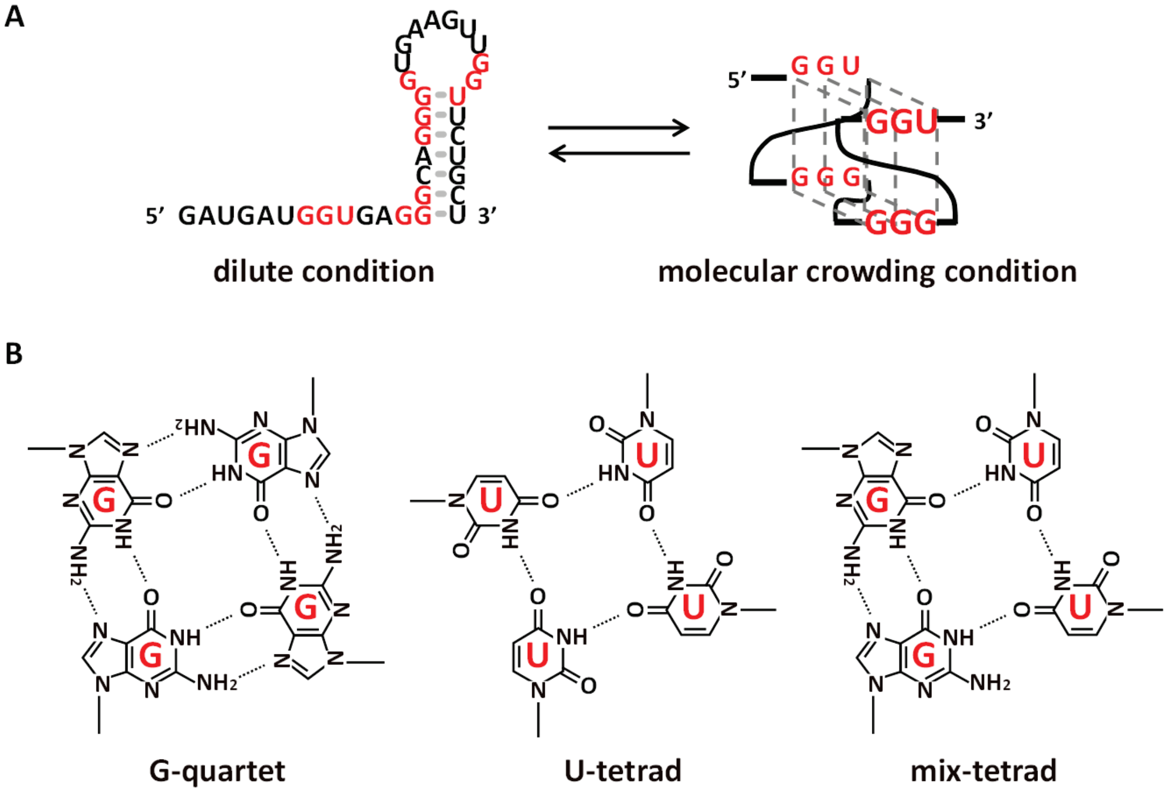

Figure S2. A) Schematic of structures adopted by the U-mutant at equilibrium. The structures based on canonical Watson-Crick base pairs (left) and G-quadruplex (right) are preferentially formed in dilute and molecular crowding conditions, respectively. B) Structures of G-quartet and U-tetrad, and mix-tetrad likely formed by the U-mutant. Dashed lines show hydrogen bond.

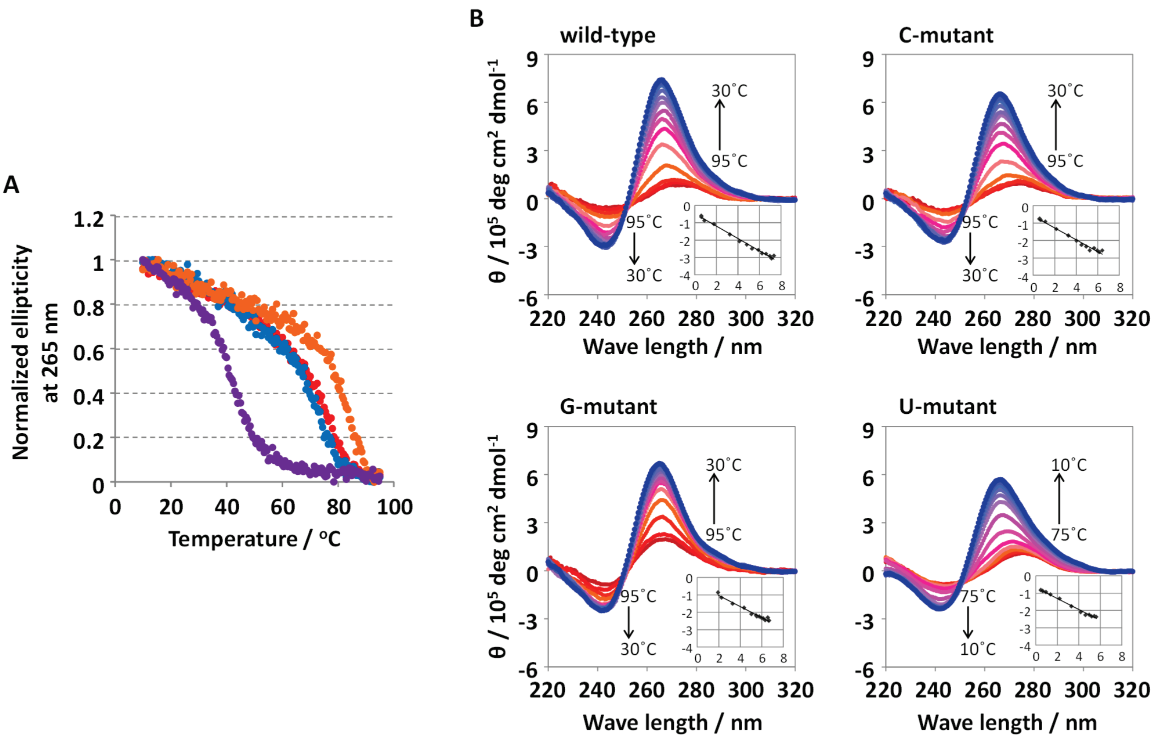

Figure S3. A) Normalized CD melting transitions at 265 nm of QFP sequence variants, wild-type (red), C-mutant (blue), G-mutant (orange), and U-mutant (purple), in a buffer containing 50 mM MES-LiOH, pH 7.0, 3 mM KCl, and 40 wt% PEG200. B) Temperature-dependent CD spectra of QFP sequence variants in a buffer containing 50 mM MES-LiOH, pH 7.0, 3 mM KCl, and 40 wt% PEG200. RNA concentrations at measurements were 10 μM except U-mutant (20 μM). The arrows represent the direction of the signal changes as the temperature is decreased at the rate of 0.2 ˚C min^{-1}. Inset shows signal intensities (10^5 deg cm^2 $dmol^{-1}$) at 265 nm (X-axis) vs. 242 nm (Y-axis).

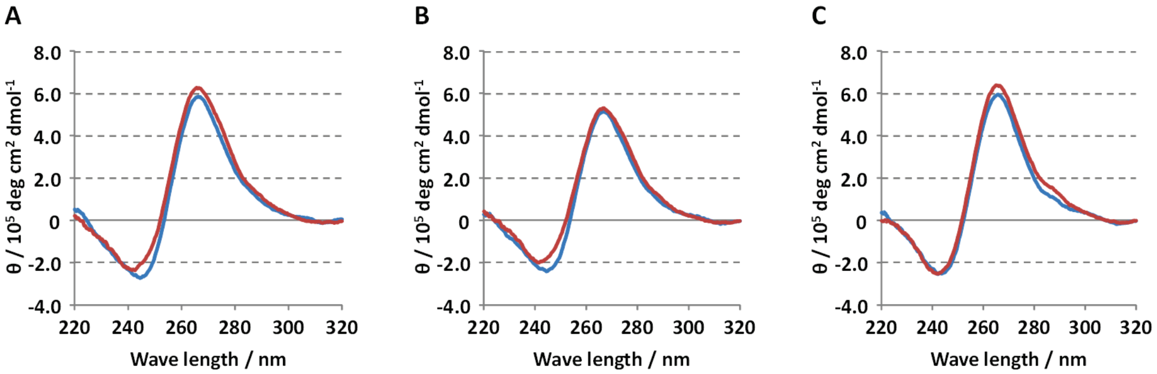

Figure S4. CD spectra of 5 μM QFP sequence variants, wild-type (A), C-mutant (B), and G-mutant (C), at 37 °C in a buffer containing 50 mM MES-LiOH, pH 7.0, and 3 mM KCl in the presence (blue) and absence (red) of 40 wt% PEG200.

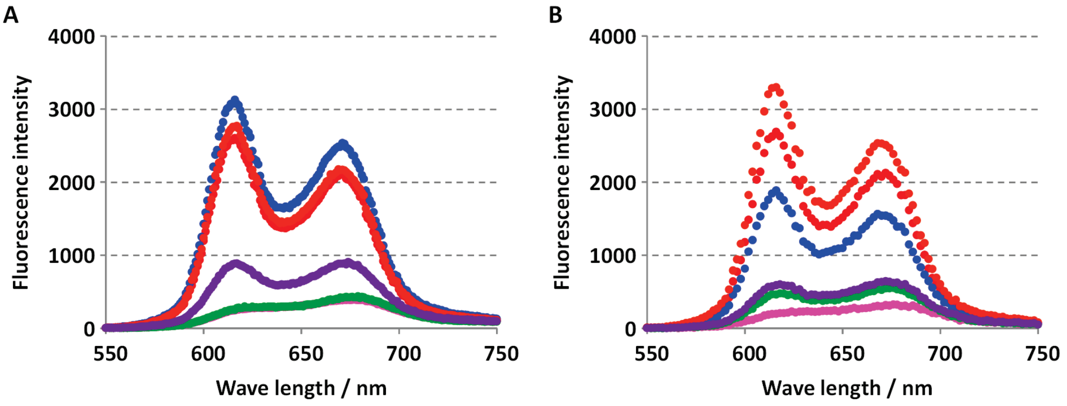

Figure S5. Fluorescence spectra of *N*-methyl mesoporphyrin (NMM) mixed with reporter mRNAs (A) or full-length *hERa* mRNAs (B) containing QFP sequence variants, wild-type (red), A-mutant (green), C-mutant (blue), G-mutant (orange), and U-mutant (purple) or without mRNA (pink). NMM (5 µM) was mixed with mRNAs (1 µM) in a buffer containing 30 mM HEPES, pH 6.8, and 100 mM KCl at 37 ˚C. Fluorescence spectra were measured from 550 nm to 750 nm with 1 nm (A) or 2 nm (B) step using 400 nm excitation. mRNAs were refolded from 90 ˚C at the rate of 1 ˚C min^{-1} before mix with NMM.

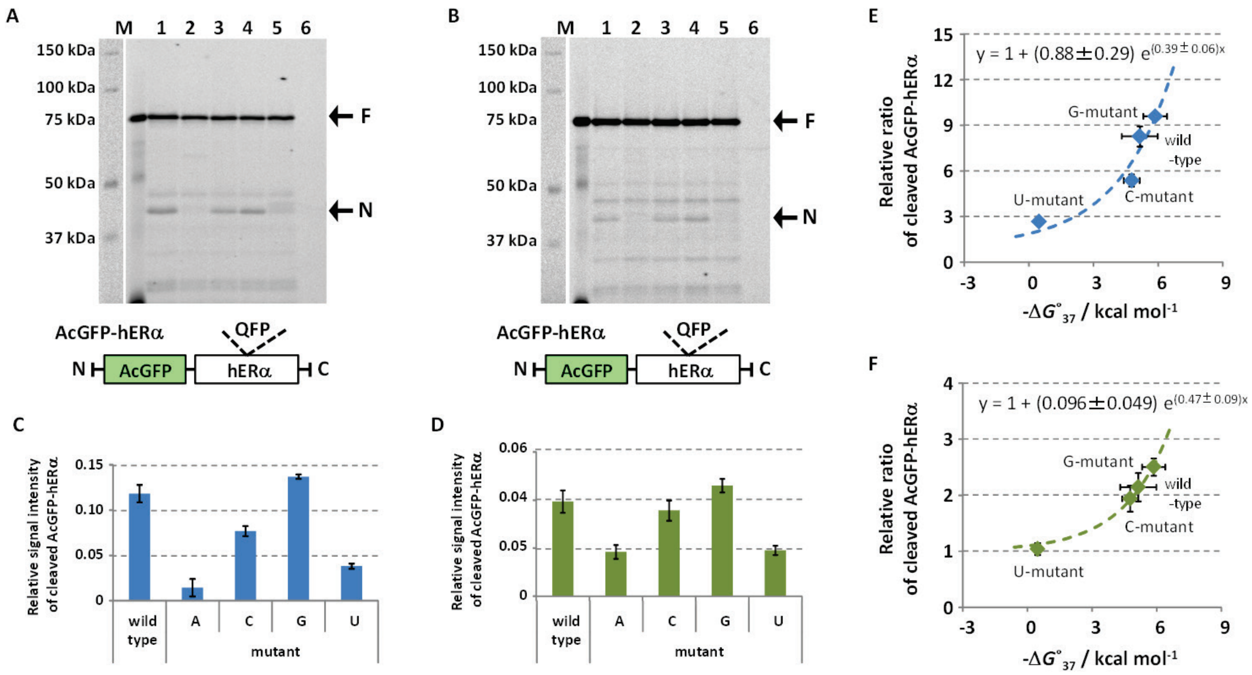

Figure S6. A, B) Protein expression patterns of AcGFP-hERα in HepG2 (A) and Flp-In 293 (B) cells. Cells were transfected with vectors for expression of AcGFP-hERα containing the QFP sequence variants, wild-type (lane 1), A-mutant (lane 2), C-mutant (lane 3), G-mutant (lane 4), and U-mutant (lane 5) or were not transfected with plasmid (lane 6). Cells were lysed after 48-h. Cell lysates were resolved on 8% SDS-PAGE without denaturing, and fluorescence signals were imaged using 473 nm excitation and 510 nm emission. C, D) Ratio of cleaved AcGFP-hERα relative to the full-length protein in HepG2 (C) and Flp-In 293 (D) cells (n = 3). E, F) Plots of the relative ratios of the cleaved AcGFP-hERα normalized to the ratio of A-mutant in HepG2 (E) and Flp-In 293 (F) *vs.* $-\Delta G°_{37}$ of the QFP sequence variants calculated from the UV melting. Fits to a single exponential equation (interior and dashed line) are indicated. Error bars represent s.d.
